# High-Efficiency and Wide-Angle Versatile Polarization Controller Based on Metagratings

**DOI:** 10.3390/ma12040623

**Published:** 2019-02-19

**Authors:** Kun Song, Ruonan Ji, Duman Shrestha, Changlin Ding, Yahong Liu, Weiren Zhu, Wentao He, Huidong Liu, Yuhua Guo, Yongkang Tang, Xiaopeng Zhao, Jiangfeng Zhou

**Affiliations:** 1 Department of Applied Physics, Northwestern Polytechnical University, Xi’an 710129, China; jiruonan@nwpu.edu.cn (R.J.); dingchanglin@nwpu.edu.cn (C.D.); yhliu@nwpu.edu.cn (Y.L.); leimu666233@163.com (W.H.); a1261843107@126.com (H.L.); 15353506120@163.com (Y.G.); guada520@mail.nwpu.edu.cn (Y.T.); 2 Department of Physics, University of South Florida, 4202 East Fowler Ave, Tampa, FL 33620, USA; duman@mail.usf.edu; 3 Department of Electronic Engineering, Shanghai Jiao Tong University, Shanghai 200240, China; weiren.zhu@sjtu.edu.cn

**Keywords:** metagratings, polarization controller, multifunction, wide-angle, dual mode

## Abstract

Metamaterials with their customized properties enable us to efficiently manipulate the polarization states of electromagnetic waves with flexible approaches, which is of great significance in various realms. However, most current metamaterial-based polarization controllers can only realize single function, which has extremely hindered the expansion of their applications. Here, we experimentally demonstrate highly efficient and multifunctional polarization conversion effects using metagrating by integrating single-structure metallic meta-atoms into the dielectric gratings. Benefiting from the combined advantages of the gratings and the metamaterials, the considered metagrating can operate in transmission and reflection modes simultaneously, acting as a high-performance and wide-angle quarter-wave or half-wave plate with distinct functions in different frequency bands. This metagrating structure is scalable to other frequency ranges and may provide opportunities to design compact multifunctional optical polarization control devices.

## 1. Introduction

As one of the key properties of an electromagnetic wave, polarization has numerous fascinating applications in the scientific research area as well as our daily life. However, limited by the intrinsic electromagnetic properties of natural materials, the conventional polarization control devices often suffer from bulky configurations, low efficiencies, and narrow working bandwidths [1], which extremely hinders their potential applications. In addition, to comply with the trend of integration and miniaturization of the electromagnetic system, integration of multiple diversified functionalities into a single and compact device has attracted increasing attention.

In recent years, the development of metamaterials opened new opportunities to efficiently manipulate electromagnetic waves [2,3,4,5,6]. With well-designed meta-atoms, metamaterials exhibit an incomparable superiority in achieving giant anisotropy or chirality which is much larger than that of natural materials [7,8,9,10,11], making it possible to control electromagnetic wave polarizations with sub-wavelength profiles [7,10,12,13,14]. On this basis, various promising ultra-compact polarization controllers based on metamaterials have been proposed, such as polarization rotators [15,16,17,18], quarter-wave plates [19,20,21,22], half-wave plates [23,24,25], asymmetric transmission devices [26,27,28], and chiral mirrors [29,30,31,32]. Despite the great progress, most of the aforementioned metamaterial-based polarization controllers possess single functionality only, which inevitably restricts the application flexibilities to some degree. Recently, considerable efforts have been devoted to integrating multiple functionalities into a single meta-device [31,33,34,35,36], and some multifunctional polarization controllers, such as multi-modal reflective metasurface polarization generator [37], metasurface with absorption and polarization conversion functions [38], metasurface with reconfigurable conversions of reflection, transmission, and polarization states [39], have been demonstrated. Additionally, some previous papers have concluded that grating structures can also realize multiple polarization conversions [40,41,42]. In spite of the described advantages, some of these polarization controllers need to integrate multifarious complex supercells to accomplish multi-functionalities [37], which are unfavorable to the structure design and fabrication process. Additionally, limited by the current design concept, these multifunctional polarization controllers work in single reflective or transmissive mode merely [37,38,39,40,41,42], and to date, a multifunctional polarization controller that can operate in dual modes, which may find important applications in multipath systems, has not yet been obtained. Thus, efficiently integrating multiple distinct polarization conversion functions and dual operating modes into a single polarization controller composed of simple geometric structures is meaningful and still a challenge. 

More recently, metagratings with excellent performances have been proposed to efficiently manipulate electromagnetic waves. For instance, Khorasaninejad et al. demonstrated that a metagrating composed of dielectric ridge waveguides can realize broadband and efficient routing (splitting and bending) into a single diffraction order and additional polarization beam splitter capabilities [43]. In another study, Ra’di et al. proposed a metagrating consisting of periodic arrays of anisotropic inclusions to control the wave front with unitary efficiency [44]. However, manipulating the polarization state of the electromagnetic waves via metagratings has not yet been reported. Inspired by the concept of metagratings, in this paper we propose a new route to manipulate the polarization state and amplitude of electromagnetic waves with an anisotropic metagrating which is obtained through the combination of metallic metamaterial and dielectric grating. Furthermore, by delicately designing the anisotropic meta-atoms, the unique metagrating is capable of realizing polarization-sensitive phase responses and can efficiently work in the transmission and reflection modes simultaneously. Both of the simulation and experimental results showed that multiple high-efficiency diversified functionalities, including linear polarization rotator, circular polarizer, circular polarization converter, and chirality preserving mirror, are integrated into this single sub-wavelength meta-device. Moreover, the corresponding efficient polarization conversion behaviors in different frequency bands are all insensitive to the incident angle of electromagnetic waves. Such a multifunctional metagrating will provide greater flexibility in a variety of practical applications where polarization controllers are involved.

## 2. Theory and Structure Design 

**Figure 1:**
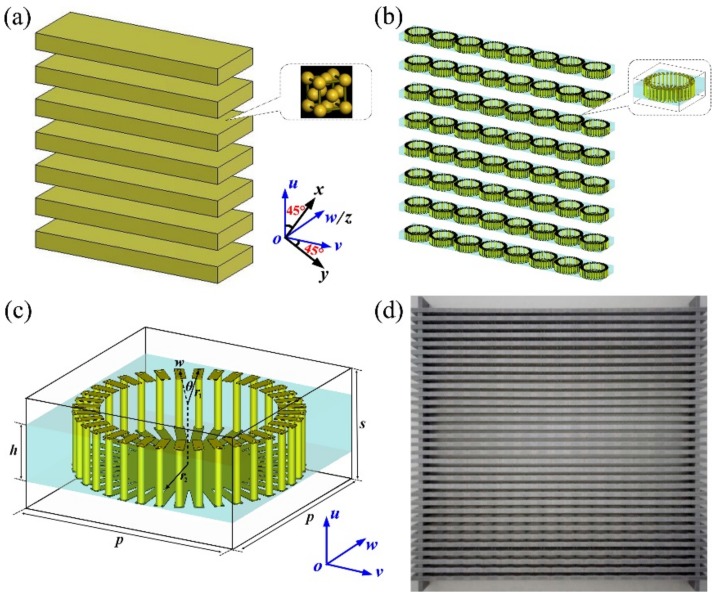
Schematic diagram of (**a**) the metallic subwavelength grating, (**b**) the proposed metagrating, and (**c**) the unit cell of metagrating. The *u*- and *v*-axis are the two principal axes of the metagrating, respectively. The direction of the *x*(*y*)-axis is rotated by 45° with respect to the *u*(*v*)-axis. (**d**) Photograph of the experimental sample.

When an anisotropic structure with the principal axis along *u*- and *v*-axis directions, for instance, a typical metal grating shown in Figure 1a, is illuminated by a plane wave propagating along *w*-axis with the polarization orientation of 45° with respect to *u*-axis direction, the reflected and transmitted electric fields can be written as:

(1) $$\left(\begin{array}{c} E_{ou}\\ E_{ov} \end{array}\right)=\left(\begin{array}{cc} a & 0\\ 0 & b \end{array}\right)\left(\begin{array}{c} E_{iu}\\ E_{iv} \end{array}\right)=a\left(\begin{array}{c} E_{iu}\\ \alpha Exp(i\beta )E_{iv} \end{array}\right),$$

where *a* and *b* are the reflection and transmission coefficients of the anisotropic structure along *u*- and *v*-axis, respectively, and the ratio of *α* = |*b*|/|*a*| is defined as the amplitude ratio. *β* is the phase difference between the two principal axes. For a perfect polarization converter, the value of *α* should be equal to 1 and the value of *β* should be ±π/2 for the quarter-wave plate and ±π for the half-wave plate.

Due to the dispersion, *β* may achieve ±π/2 and ±π at different wavelengths, thereby a single device can exhibit multifunctional polarization conversion as both quarter-wave and half-wave plates [40,41]. However, such multifunctional wave plates are only able to operate within a narrow band due to the dispersion nature. 

In this work, we choose the grating structure to achieve multiple polarization conversion because of its simultaneous control of phases and amplitudes capabilities [40,41,42,45]. Benefiting from the concept of metamaterials, we are able to control *α*, *β*, and even operating modes through a more flexible approach by integrating artificial meta-atoms into conventional gratings. Here, as shown in Figure 1b, we propose a sub-wavelength metagrating composed of single wheel-like unit cells as an example. Figure 1c exhibits the detailed geometrical structure of the unit cell. The bilayer metallic patterns are connected by a metallization hole with the diameter of 0.6 mm. The structural parameters of the unit cell are as follows: *p* = 12 mm, *s* = 6 mm, *r*_1_ = 5.9 mm, *r*_2_ = 3.5mm, *w* = 0.5 mm, *h* = 3 mm, and *θ* = 10°. The metal cladding is copper with the thickness of 0.035 mm and conductivity of *σ* = 5.8 × 10^7^ S/m. The dielectric substrate is Teflon with a relative permittivity of 2.2 + 0.001 × *i*. Figure 1d shows the photograph of the fabricated metagrating structure, which is composed of 33 layers of slats each consisting of 16 wheel-like unit cells.

## 3. Results and Discussion

In order to get the electromagnetic properties of the proposed metagrating, both of the simulations and experiments were carried out. The simulations were achieved with the commercial finite element software CST Microwave Studio (CST2018, Computer Simulation Technology GmbH). In the simulations, unit cell boundary conditions were used in the *u*- and *v*-axis directions, while open boundary conditions were employed in the *w*-axis direction. The experimental data was obtained using a network analyzer (AV3629, 41st institute of CETC, Qingdao, China) with two broadband linearly polarized horn antennas in an anechoic chamber.

**Figure 2:**
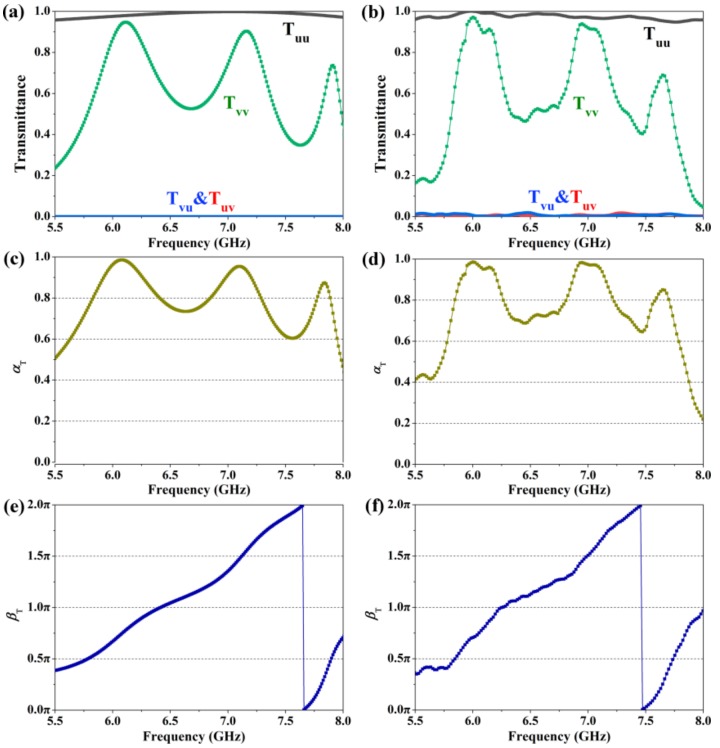
Simulation (left column) and experimental (right column) results of the proposed metagrating in the case of *u*- and *v*-polarization incidence. In this frequency region, the metamaterial operates in transmission mode. (**a**,**b**) Transmittance, (**c**,**d**) calculated amplitude ratio *α*_T_, (**e**,**f**) phase difference *β*_T_ between *u*- and *v*-axis.

Since the considered metagrating can work in the transmission and reflection modes at the same time, we first studied the transmission mode of the metagrating in the frequency range of 5.5 GHz to 8.0 GHz. Figure 2 shows the transmittance, amplitude ratio, and phase difference of the proposed metagrating as the linearly polarized incident waves are *u*- and *v*-polarizations. In this paper, the first subscript *m* in *T*_mn_/*R*_mn_ represents the polarization state of the transmitted and reflected wave, while the second one *n* represents the incident wave. As shown in Figure 2a, the co-polarization spectrum (*T*_vv_) of *v*-polarization incident waves shows there are three peaks with high transmittance occurring on the co-polarization spectrum (*T*_vv_) of *v*-polarization incident waves. For the *u*-polarization incident waves, the metagrating will operate similarly as a regular wire-grid metallic grating, which enables a high transmittance for the linearly incident waves with the polarization plane perpendicular to its wire line direction [18,46], leading to a high co-polarization transmittance (*T*_uu_) over 0.95 in the whole frequency range. Since the transmittance of *T*_uu_ and *T*_vv_, are close to each other around the frequencies of 6.1 GHz, 7.1 GHz, and 7.9 GHz, the amplitude ratios *α*_T_ = *T*_vv_/*T*_uu_ in Figure 2c are close to 1 around the corresponding frequencies, which is important to ensure a perfect quarter-wave or half-wave plate performance. Figure 2e portrays the transmission phase difference *β*_T_ between *u*- and *v*-axis (*β*_T_ = *φ*_vv_ − *φ*_uu_). It is obvious that the phase difference *β*_T_ can be varied in a large dynamic range covering from 0 to 2π. And it can also be found that around the frequencies of 5.8 GHz, 6.4 GHz, 7.1 GHz, and 7.9 GHz, the phase differences are approximately about π/2, π, 3π/2, and π/2, respectively, which implies that the metagrating is able to function as a transmission-type half-wave or quarter-wave plate at the selected frequencies. The experimental results of the proposed metagrating are shown in Figure 2b,d,f, respectively, which show good agreement with simulations.

**Figure 3:**
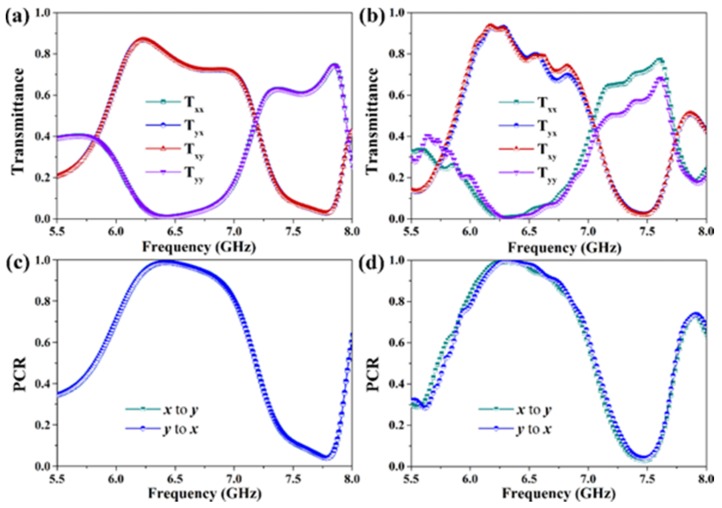
Simulated (left column) and measured (right column) results of the designed metagrating for the *x*- and *y*-polarized incident waves in transmission mode. (**a**,**b**) Transmittance, (**c**,**d**) PCR.

In Figure 3, we illustrate the results of the considered metagrating in the case of *x*- and *y*-polarized wave incidence. It is significant that in Figure 3a the co-polarization and cross-polarization transmission spectra for the *x*- and *y*-polarized incident waves are completely coincident, i.e., *T*_xx_ = *T*_yy_ and *T*_yx_ = *T*_xy_. This phenomenon can be attributed to the unit cell that is mirror symmetry with respect to the plane *x* + *y* = 0 (*uow* plane) [23]. In the frequency range from 5.8 GHz to 7.1 GHz, the cross-polarized waves account for the majority of transmitted waves, and the highest transmittance of the cross-polarization rise up to 0.87 at 6.2 GHz, which means that as an *x*(*y*)-polarized incident wave passes through the proposed metagrating, the transmitted wave is mainly transformed into *y*(*x*)-polarized wave around this frequency. While in the frequency range of 7.1 GHz to 7.9 GHz, the co-polarized waves dominate the transmitted waves, and the highest transmittance is 0.75 at 7.8 GHz. As for the intersections of co-polarization and cross-polarization transmission curves, the metagrating will work as a quarter-wave plate. According to the phase differences *β*_T_ shown in Figure 2e,f, in the frequency vicinity of 5.8 GHz, 7.1 GHz, and 7.9 GHz, the transmitted waves are left-handed (right-handed), right-handed (left-handed), and left-handed (right-handed) circular polarizations with the transmittance over 0.78 for an *x*(*y*)-polarized incident wave, respectively. Particularly, the transmittance of the right-handed circularly polarization wave is nearly close to 1 at 7.1 GHz, indicating a perfect linear-to-circular polarization conversion effect. In Figure 3c, the polarization conversion ratio (PCR), which is defined as PCR = *T*_cross_/(*T*_cross_ + *T*_co_), is used to characterize the polarization conversion efficiency of the metagrating. It shows that the PCR is larger than 0.9 between 6.1 GHz and 6.8 GHz and reaches its maximum value of 0.99 at 6.4 GHz, where the metagrating will operate as a nearly perfect half-wave plate. The experimentally measured results are respectively plotted in Figure 3b,d, which exhibit similar phenomena as the simulations in spite of the slight deviations. According to the aforementioned results, the proposed metagrating is capable of acting as a high-performance multiband transmission-type wave plate.

**Figure 4:**
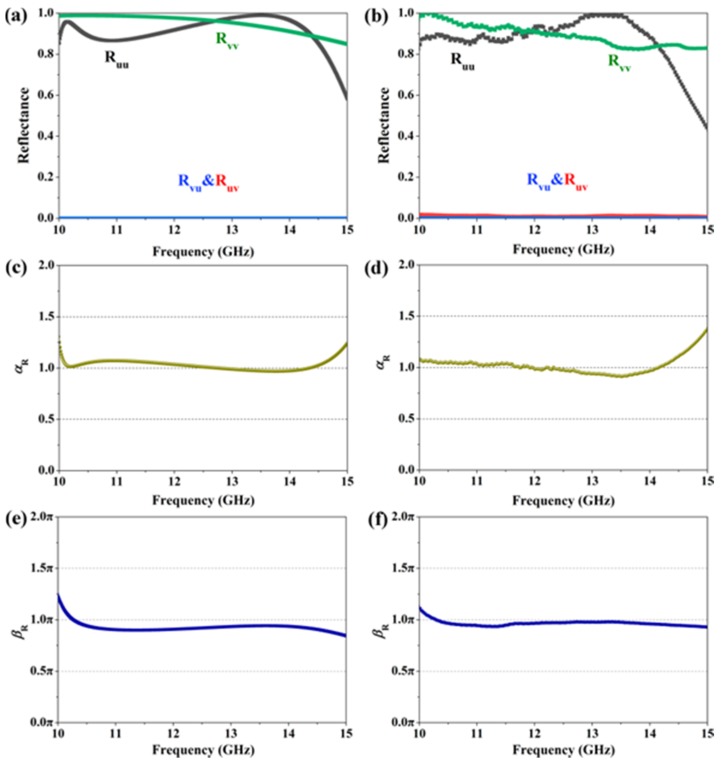
Numerical (left column) and experimental (right column) results of the proposed metamaterial under *u*- and *v*-polarization incidence in the reflective frequency range. (**a**,**b**) Reflectance, (**c**,**d**) amplitude ratio *α*_R_, (**e**,**f**) phase difference *β*_R_ between *u*- and *v*-axis.

Between 10.0 GHz and 15.0 GHz, the intriguing metagrating will operate in reflective mode. Figure 4 plots the results of the proposed metagrating for *u*- and *v*-polarized wave incidence. In Figure 4a,b, it is seen that the metagrating can realize high reflection for both of *u*- and *v*-polarized incident waves, with the simulated (experimental) reflectance of *R*_uu_ and *R*_vv_ 0.85 (0.83) from 10.0 GHz to 14.5 GHz. The corresponding results of the amplitude ratios *α*_R_ are shown in Figure 4c,d, separately. Obviously, the values of *α*_R_ are approximately 1 in this frequency range. Additionally, it can be found from Figure 4e,f, that the phase differences of reflection waves along the two principal axes are roughly maintained π in the frequency range of 10.0 GHz to 14.5 GHz. And the features mentioned above enable the metagrating to act as a broadband reflection-type half-wave plate with high efficiency.

**Figure 5:**
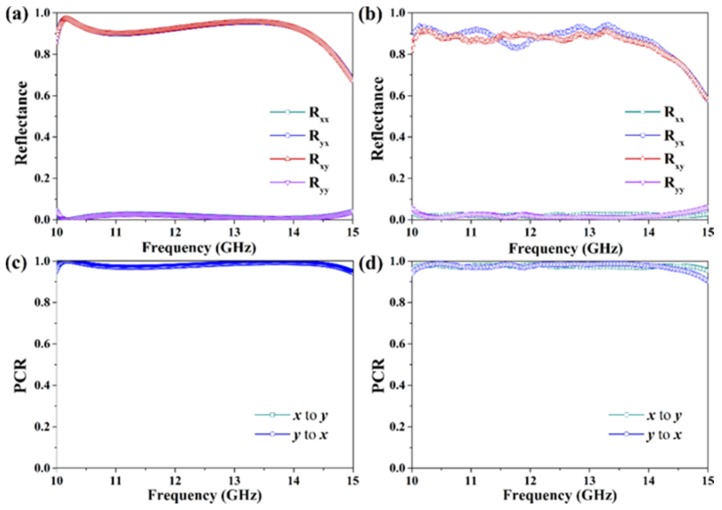
Simulation (left column) and experimental (right column) results of the proposed metagrating for the *x*- and *y*-polarized incident waves in the reflective mode. (**a**,**b**) Reflectance, (**c**,**d**) PCR.

Figure 5 shows the reflective spectra and the corresponding PCR of the designed metagrating for the *x*- and *y*-polarized incident waves. As shown in Figure 5a,b, the co-polarization and cross-polarization reflection spectra for the *x*-polarized incident wave are as same as those of *y*-polarized incident wave, respectively. In the frequency range of 10.0 GHz to 14.3 GHz (the relative bandwidth is about 35.4%), the reflectance of cross-polarization reflection spectra, *R*_yx_ and *R*_xy_, are larger than 0.9, while those of the co-polarization reflection spectra, *R*_xx_ and *R*_yy_, are less than 0.03. This fact reveals that, for an *x*(*y*)-polarized incident wave, the reflected wave will be efficiently converted to be *y*(*x*)-polarized wave. In Figure 5c,d, we further calculate the PCR of the reflection waves of the metagrating. It is significant that both of the simulated and measured PCR are over 0.95 from 10.0 GHz to 14.3 GHz, exhibiting a nearly perfect reflective cross-polarization conversion effect. Therefore, the considered metagrating is able to work as a high-performance reflection-type half-wave plate in a broad bandwidth.

**Figure 6:**
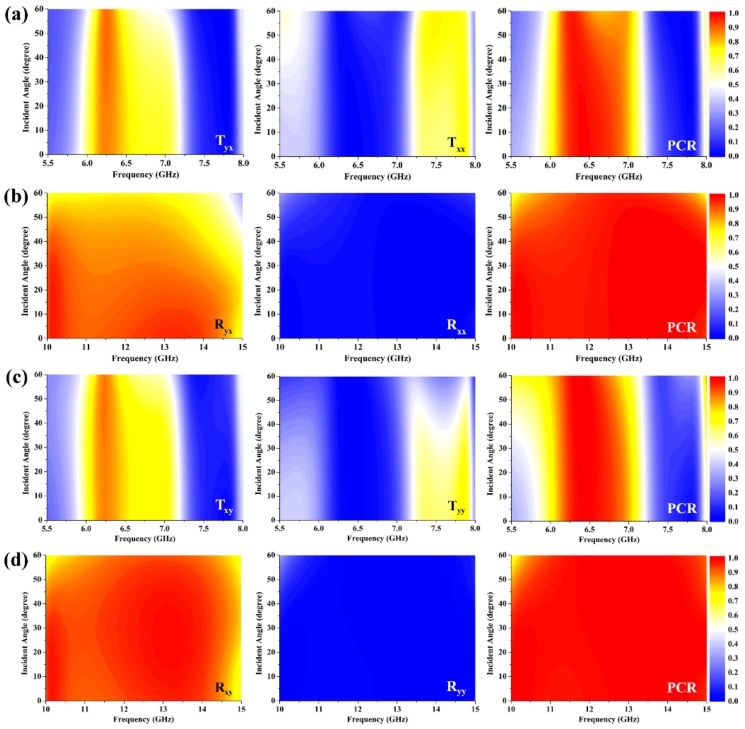
The effects of incident angle on the electromagnetic properties of the metagrating for the linearly polarized incident waves. The results of the metagrating in (**a**) transmission and (**b**) reflection modes under *x*-polarization incidence. The results of the metagrating in (**c**) transmission and (**d**) reflection modes under *y*-polarization incidence. At oblique incidence, the *x*- and *y*-polarization waves actually represent the TM and TE waves, respectively. In the simulations, the incident angle is increasingly tuned by a step of 5°.

The electromagnetic properties of the metagrating for the linearly polarized waves at oblique incidence were also studied, as shown in Figure 6. Figure 6a shows the influences of incident angle on the cross-polarization spectra, co-polarization spectra, and PCR of the metagrating in transmission mode for the *x*-polarized incident wave. Owing to the isotropic design of the unit cell, our metagrating shows consistent performance as the incident angle varies within a large range. As the incident angle increases, the transmittance of co-polarization and cross-polarization transmission at 5.8 GHz and 7.9 GHz both slightly increase, while a slight reduction phenomenon occurs at 7.1 GHz. Despite all this, the considered metagrating can still work well as an excellent quarter-wave plate with high transmittance over 0.8 at these frequencies even if the incident angle rises up to 40°. Additionally, it is worth noting that the variation of incident angle has almost no effect on the maximum transmittance at 6.2 GHz, and the corresponding PCR remain as high as 0.95 when the incident angle is 60°, implying a highly efficient and wide-angle half-wave plate function. In Figure 6b, it is seen that the co-polarized reflectance increases slightly as the incident angle increases, while the cross-polarized reflectance gradually decreases. In spite of the decrement, the cross-polarized reflectance is still larger than 0.8 from 10 GHz to 13.8 GHz as the incident angle is 40°, simultaneously accompanied by a PCR over 0.9. Hence, the broadband and efficient reflective cross-polarization conversion effect of the metagrating is insensitive to the incident angle of the *x*-polarized wave. In the case of *y*-polarization incidence, a similar polarization conversion of the transmission can also be observed, although the transmittance of the right-handed circularly polarized wave was decreased at around 5.8 GHz. More interestingly, the reflection mode of the metagrating exhibits a much stronger cross-polarization conversion with a wider bandwidth and a lager range of angle invariance in comparison to the case of *x*-polarization incidence, as shown in Figure 6c,d. Thus, the intriguing metagrating can function as a dual operating mode, high-performance, and multiple functional wave plate regardless of the incident angle, which exhibits more flexibility than the previous metamaterial-based wave plates [15,16,17,23,25].

As the designed metagrating is anisotropic, it can also operate for the circularly polarized wave incidence indeed. For the circular polarization basis, the circularly polarized transmission and reflection coefficients can be respectively obtained via the linear ones by the following equations [2,30]:

(2) $$\left(\begin{array}{cc} t_{++} & t_{+-}\\ t_{-+} & t_{--} \end{array}\right)=\frac{1}{2}\left(\begin{array}{cc} t_{xx}+t_{yy}+i(t_{xy}-t_{yx}) & t_{xx}-t_{yy}-i(t_{xy}+t_{yx})\\ t_{xx}-t_{yy}+i(t_{xy}+t_{yx}) & t_{xx}+t_{yy}-i(t_{xy}-t_{yx}) \end{array}\right)$$

(3) $$\left(\begin{array}{cc} r_{++} & r_{+-}\\ r_{-+} & r_{--} \end{array}\right)=\frac{1}{2}\left(\begin{array}{cc} r_{xx}+r_{yy}+i(r_{xy}-r_{yx}) & r_{xx}-r_{yy}-i(r_{xy}+r_{yx})\\ r_{xx}-r_{yy}+i(r_{xy}+r_{yx}) & r_{xx}+r_{yy}-i(r_{xy}-r_{yx}) \end{array}\right)$$

Here, the subscripts ‘+’ and ‘–’ represent the clockwise and counterclockwise circularly polarized waves when separately observed along +*z* direction. As the wave vectors are in opposite directions for transmitted and reflected circularly polarized waves, one should notice that each reflection component of the circularly polarized waves has different physical meaning compared with the transmission case. Thus, the transmission coefficients are defined as $t_{RR}=t_{++}$, $t_{LL}=t_{--}$, $t_{LR}=t_{-+}$, $t_{RL}=t_{+-}$, while the reflection coefficients are defined as $r_{LR}=r_{++}$, $r_{RR}=r_{-+}$, $r_{LL}=r_{+-}$, $r_{RL}=r_{--}$. Additionally, $t_{RR}$, $t_{RL}$, $r_{RR}$, and $r_{RL}$ represent the right-handed circularly polarized (RCP) waves, while $t_{LL}$, $t_{LR}$, $r_{LL}$, and $r_{LR}$ indicate the left-handed circularly polarized (LCP) waves.

According to the transmittance in Figure 3a and the reflectance in Figure 5a, we can easily deduce that the transmission and reflection coefficients satisfy the relationships of $t_{xx}=t_{yy}$, $t_{yx}=t_{xy}$, $\;r_{xx}=r_{yy}$, and $r_{yx}=r_{xy}$ at normal incidence, thus the Equations (2) and (3) can be further simplified as:

(4) $$\left(\begin{array}{cc} t_{++} & t_{+-}\\ t_{-+} & t_{--} \end{array}\right)=\left(\begin{array}{cc} t_{xx} & -it_{xy}\\ it_{xy} & t_{xx} \end{array}\right),$$

(5) $$\left(\begin{array}{cc} r_{++} & r_{+-}\\ r_{-+} & r_{--} \end{array}\right)=\left(\begin{array}{cc} r_{xx} & -ir_{xy}\\ ir_{xy} & r_{xx} \end{array}\right).$$

Namely, the transmittance and reflectance of the circularly polarized wave satisfy $T_{LL}=T_{RR}=T_{xx},\;\;T_{RL}=T_{LR}=T_{yx},\;\;R_{LL}=R_{RR}=R_{xy},\;\;R_{RL}=R_{LR}=R_{xx}$. 

**Figure 7:**
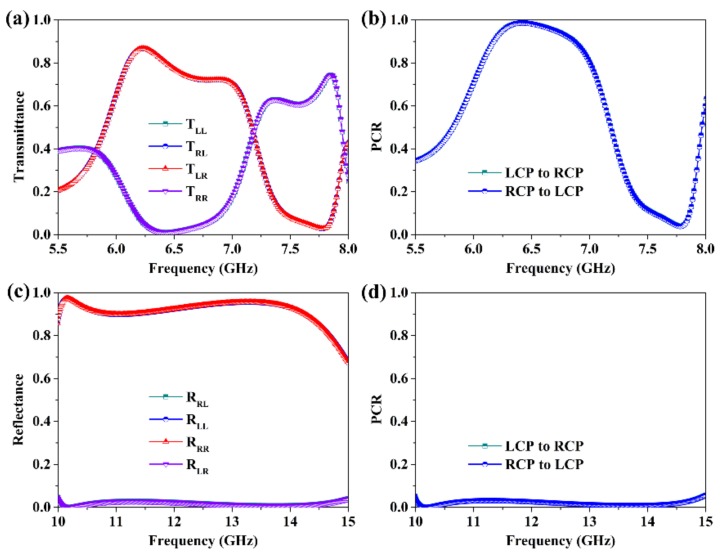
Simulation results of the designed metagrating for the circularly polarized waves at normal incidence. (**a**) Transmittance and (**b**) PCR spectra in transmission mode, (**c**) reflectance and (**d**) PCR spectra in reflection mode.

In Figure 7, we illustrate the simulated results of the metagrating for the circularly polarized waves at normal incidence. It is obvious that the transmittance and PCR spectra for the circularly polarized incident waves shown in Figure 7a,b, respectively, are completely the same as the ones in Figure 3. These phenomena can be well explained by Equations (2) and (4). It should be noted that, for the LCP (RCP) incident waves, the transmitted waves are *x*(*y*)-polarized, RCP (LCP), *y*(*x*)-polarized, and *x*(*y*)-polarized at 5.8 GHz, 6.4 GHz, 7.1 GHz, and 7.9 GHz, respectively. Figure 7c plots the reflectance spectra of the proposed metagrating. It can be found that as a circularly polarized wave is incident on the metagrating, the co-polarization reflectance ($\;R_{LL}$ or $R_{RR}$) is larger than 0.9 from 10.1 GHz to 14.3 GHz, while the cross-polarization reflectance ($\;R_{RL}$ or $R_{LR}$) is less than 0.03. That is to say, our design can function as a nearly perfect chirality preserving mirror in a broad bandwidth, consequently leading to a very low PCR of less than 0.03, as shown in Figure 7d. 

**Figure 8:**
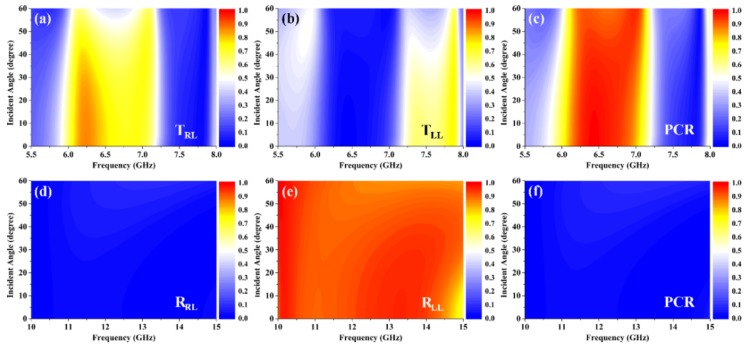
Influences of incident angle on the electromagnetic properties of the metagrating in the case of circular polarization incidence. (**a**) Cross-polarization transmittance, (**b**) co-polarization transmittance, and (**c**) PCR in transmission mode; (**d**) cross-polarization reflectance, (**e**) co-polarization reflectance, and (**f**) PCR in reflection mode.

Figure 8 shows the influences of incident angle on the electromagnetic properties of the proposed metagrating in the case of circular polarization incidence. It can be seen in Figure 8a–c, that in transmission mode the operating frequencies generate slight blue shifts as the incident angle increases. Although the values of the cross-polarization transmittance gradually reduce as the incident angle increases, the highest transmittance of the cross-polarization can still be maintained over about 0.8 when the incident angle increases to 40°, simultaneously accompanied by a high PCR larger than 0.9. In Figure 8d–f, it is shown that the effects of incident angle on the co-polarization and cross-polarization reflectance spectra are inappreciable. And the lowest value of co-polarization reflectance is still over about 0.9 in the frequency range of 10.0 GHz to 14.3 GHz even when the incident angle is 40°, while the cross-polarization reflectance is no more than 0.05 with the corresponding PCR being less than 0.05. The aforementioned results further confirm that the proposed metagrating can also act as a dual-mode, wide-angle, and multifunctional wave plate with high-efficiency for the circularly polarized incident waves. 

## 4. Conclusions

In summary, we have proposed an effective way to achieve multifunctional polarization manipulation by a metagrating consisting of periodically arranged meta-atoms inside dielectric gratings. As a proof-of-concept experiment, we have designed and fabricated a sub-wavelength metagrating that consists of wheel-like meta-atoms. The metagrating works in both transmission and reflection modes efficiently. The phase differences between the two principal axes are monotonically altering with the frequencies in transmission mode covering a large dynamic range from 0 to 2π, while they are approximately kept at about π in a broad bandwidth region in reflective mode. Our measurements show that the metagrating can work as a dual-mode, high-efficiency, and multifunctional wave plate to realize various functions including linear-to-circular polarization conversion, linear or circular cross-polarization conversion, and chirality preserving mirror in different frequency bands simultaneously accompanied by a large angular invariance. Such versatile functionalities provide great flexibilities for optical polarization control devices. The design of metagrating can be extended to other frequency ranges as a compact optical polarization controller in the applications of telecommunications, radar detections, and optical devices.
